# A Hospital as a Cinema Theatre

**Published:** 1912-03-02

**Authors:** 


*To the Editor of* The Hospital.

A HOSPITAL AS A CINEMA THEATRE.

*To the Editor of* The Hospital.

Sir,—In reply to a letter signed "Lex" in your issue of February 24, I particularly pointed out in my previous letter that the remarks in your issue dated February 10 were misleading "so far as London is concerned." The Cinematograph Act in London is administered by the County Council, and the provisions of it must be considered *inter alia* with the other several Acts having reference to public buildings within the area of the county.

Though the expression "public building" does not occur in the Cinematograph Act its administration is vested in the County or Borough Authority, and the building laws of the particular district would determine the "class" of the building. In short, no building can be erected or adapted to be used for the permanent purpose of a Cinematograph Theatre having regard only to the Cinematograph Act. I am, Sir, etc.,

London, February 27. District Surveyor.

